# Integrating High-Impact Practices (HIPs) In Medical Curriculum At Northern Border University: Strengths, Challenges, and Examples

**DOI:** 10.7759/cureus.39938

**Published:** 2023-06-04

**Authors:** Anshoo Agarwal, Anil M Rao, Awdah M Alhazimi, Mohammed M Ismail, Eslam K Fahmy

**Affiliations:** 1 Pathology, College of Medicine, Northern Border University, Arar, SAU; 2 Pathology, Faculty of Medicine, Northern Border University, Arar, SAU; 3 Physiology, Faculty of Medicine, Northern Border University, Arar, SAU; 4 Anatomy, Faculty of Medicine, Northern Border University, Arar, SAU; 5 Medical Education and Physiology, Faculty of Medicine Northern Border University, Arar, SAU

**Keywords:** high impact practices (hips), medical students, learning outcomes, curriculum design, undergraduate

## Abstract

Background: High-Impact Practices (HIPs) are educational practices that have been shown to increase rates of student retention, engagement, and persistence to graduation which help them to become high achievers and lifelong learners. Universities strongly encourage faculty members to incorporate one or more of these HIPs in order to improve active learning among students. Students are met with a variety of experiences that are not entirely of their choice, including expectations for academic performance, interactions with faculty, staff, and peers, and extracurricular activities that may or may not match their expectations and skills. Higher retention and high-grade achievement rates are attributed to HIPs. The mechanism by which HIPs improve retention is poorly understood.

Aims and objectives: There are numerous analyses of the objectives particular to undergraduate medical education in recent years. There have been proposed three major target categories. Undergraduate medical education has been positioned within a liberal education framework, where the main objective is to equip students with the critical thinking abilities, broad general education, and subject-specific knowledge they will need to be able to effectively problem-solve, adapt to new roles, and apply public health thinking and practices to a variety of situations. We tried to incorporate HIPs in a medical curriculum at the Faculty of Medicine, Northern Border University, by giving them topics that can be used to create public awareness about the selected objectives which may help the community greatly.

Methodology: Students were asked to make posters or videos on the topics and were asked to write reflections about their experience and give feedback to the coordinators for improvements and to make these HIPs better so that they can be included uniformly in the other courses as well.

Results and conclusions: Based on results from a random sample of undergraduate students, we draw the conclusion that HIPs are correlated with engagement, which is the alignment of the student's critical thinking and ability to work in effective teams, group projects, learning communities, and sequence courses. HIPs have an impact on involvement among students across the world. HIPs are effective to the extent that they engage pupils, encouraging a greater commitment, which is one way to understand their success.

## Introduction

High-impact practices (HIPs) are scholastic practices that have been shown to increase rates of student retention, engagement, and persistence to graduation which help them to become high achievers and lifelong learners. Rather, they are effective teaching methods that cater to students' achievement both during and after the course. Universities strongly encourage faculty members to incorporate one or more of these HIPs in order to improve active learning among students. Examples of high-impact practices include seminars, writing-intensive courses, collaborative assignments and projects, service learning, capstone courses, and ePortfolios.

The transitional economies of many countries show the greatest delays in reorganizing education systems to keep up with societal and economic changes. The backlog of reforms could slow growth; nevertheless, timely reform can benefit the economy and reduce poverty, as shown by East Asian nations, who typically make sufficient investments in the development of human capital [1]. In Saudi Arabia, medical education has undergone tremendous change. A push for improved quality assurance has been linked to quantity development in medical education.

High-Impact Educational Practices (HIEPs) implementation has been recommended at higher education institutions to improve student learning, increase retention rates, and boost student engagement [2]. Increasing demand for skill development, securing necessary resources, and meeting the country's employment requirements are all issues that the education sector is currently dealing with [3]. Four phases were used to produce a set of HIPs core competencies that ought to be included in teaching and learning programs: (1) development of an initial set of pertinent competencies derived from a systematic review of education studies for health professionals; (2) a two-round, web-based survey of health professionals chosen using purposive sampling; (3) consensus meetings, both in-person and via video conference, to finalize the consensus on the most essential core competencies; and (4) feedback [4]. Colleges and universities have the problem of managing and avoiding student dropout. The problem of education abandonment affects more than just institutions of higher learning. When students stop attending courses altogether in addition to exhibiting disruptive behaviors or disinterest in the classroom, it becomes a legitimate and expensive concern. So, the retention process for adolescents and young adults entails a complex stake, one that is more provocative for the educational institution [5]. Lectures, practical sessions, problem-based learning (PBL), clinical skill sessions, clinical bedside teaching, and work-based learning in hospitals and operating rooms are just a few of the approaches used to teach medical students.

Health education seeks to alter and encourage societal health behaviour. Many of Saudi Arabia's medical schools have incorporated patient health education as a mandatory part of medical courses and are trying to incorporate high-impact or engaged learning practices as teaching strategies [6-7].

High-impact practices (HIPs) or engaged learning practices

Some methods of instruction might be more effective for students at specific levels, while others might be more suited to a particular discipline or course. Still, additional strategies and approaches might simply be more compatible with an individual instructor's teaching style. There are various teaching methods, workouts, and resources available to educators, all of which have the potential to be helpful in a particular teaching scenario. However, research suggests that some instructional approaches may have a more significant impact than others [8]. Additionally, considering that both teachers and students are frequently under time constraints, it is logical to devote particular attention to teaching strategies that provide "more value for the buck." Eleven practices have been identified as high-impact practices: (1) First-year encounters, (2) Typical intellectual experiments, (3) Educational communities, (4) Courses that focus on writing, (5) Group projects and assignments, (6) Undergraduate research, (7) Diversity/global learning, (8) Community-based learning, (9) Internships, (10) Capstone projects and courses, and (11) e-Portfolios.

The importance of "deep approaches" to learning for students cannot be overstated because they "tend to earn higher grades and retain, integrate, and transfer information at greater levels." Now the question is, what, however, qualifies as a "high impact" practice?

Characteristics of a high-quality, high-impact practice

Eight traits are listed here that when applied to various academic activities, can interest students and significantly affect their learning: (1) Set performance expectations at appropriately high levels, and effectively communicate these expectations to students; (2) Encourage students to invest significant and meaningful time and effort on authentic, complex tasks over an extended period of time; (3) Add meaningful interactions among students and between faculty and students about substantive matters; (4) Challenge students’ ways of thinking, increase interactions with individuals with experiences and life experiences different from their own experiences with diversity; (5) Provide frequent, timely, and constructive feedback; (6) Increase periodic, structured opportunities to reflect and integrate learning; (7) Provide opportunities to discover the relevance of learning through real-world applications, or add a real-world/authentic experience; (8) Add a public demonstration of competence.

These distinctive characteristics of high-impact practices need not simply apply to the conducts listed above that are frequently regarded as high-impact practices. Instead, these characteristics might serve as benchmarks for directing the planning and implementation of mandated educational opportunities customized for curricula in the Institutes. This should be explicitly taken into account in the evaluation of all educational activities since it has the potential to enhance and intensify the impacts of deep learning and engagement, with specific advantages for student populations.

A number of unresolved issues with regard to institutional and curriculum support, the conceptualization of generic competencies, teaching methods and assessment, as well as teachers' and students' perspectives, are involved in the development of generic competencies in the curricula of higher education.

Using a critical analysis of the impact of high-impact practices may give an insight demonstrates how the implementation of a shared conceptual framework may enhance teaching pedagogy, curriculum, students' experiences and learning strategies, conception, higher education missions, and compliance to get aligned to ensure the effective development of generic skills and competencies [9]. There have been numerous analyses of the learning objectives particular to undergraduate medical education in recent years [10]. There have been proposed three major target categories. In the first place, undergraduate medical education has been placed within a liberal education framework, where the main goal is to give students the critical thinking, skills, broad general education, and subject-specific knowledge they will need to be able to effectively problem-solve, adapt to new roles, and apply public health thinking and practices to a variety of situations [11]. Higher impact practices may assist in preparing students with the ability to collaborate in some way in order to enhance community health [12-13].

Another framing addresses undergraduate medical education in a professional education framework with the primary goal of educating students to develop the skills needed to be successful in the patient care workforce at the bachelor's level or to have the foundation of training to be successful in the workforce at higher levels with additional master's or doctoral-level training. The third objective is to equip students with a medical bachelor's degree with the skills necessary to choose academic graduate and professional medical career routes and training in the health professions.

High-impact practices (HIPs) have the potential to improve the standards, efficacy, and outcomes of undergraduate students learning. While some high-impact activities are purposefully incorporated into the overarching goals, learning objectives, and curricular competencies for undergraduate students, others just simply fit in. However, careful effort across the stages of curriculum design, course delivery, and program administration is required to maximize the benefits of HIPs for medical undergraduate students. We cover both conceptual frameworks and concrete procedures for incorporating HIPs into curriculum development and implementation.

## Materials and methods

In this cross-sectional study conducted at Northern Border University, third-year Bachelor of Medicine and Bachelor of Surgery (MBBS) students were included to participate in the survey. A pre-validated self-designed survey questionnaire based on the published data was administered. HIPs adopted by us included a selection of topics based on the learning objectives covered in the cardiovascular module in the medical curriculum which could be useful to create awareness in the community and may be able to help the community greatly. Students were asked to make posters or videos on the topics (which can be used for public awareness platforms like exhibitions etc). Students were asked to write reflections about their experience and give feedback to the coordinators to improve these HIPs. The integration of HIPs into the cardiovascular (CVS) Module was done based on the following key components which are given as a requirement for HIPs (Appendix 1).

(1) Challenge: Selection of topics and the task given to the students (a) to make learning objectives for their topics working in a team, (b) each student chooses one objective and works on it, (c) presents it in a power-point format or can present in poster format as a team compiling all students objectives of their respective topics.

(2) Time: (a) Before coming to the first HIPs session, students need to work in the student activity time or in their free time within their groups or can take guidance from their group facilitators by contacting them. To make learning objectives for their topics working in a team, each student needs to choose one objective and work on it interactively (read, analyze, brainstorm, identify a problem, research and design a problem). (b) At the first HIPs session, students present problems and solutions using a presentation on the chosen topics in a power-point format/video or a poster (format) compiling all students' objectives of their respective topics as a team. (c) In the second HIPs session feedback is collected via a Google form.

(3) Writing self-reflection on topics presented by the students: Reflection allows communicating about how a specific article, lesson, lecture, or experience shapes understanding and helps in learning more deeply. Reflection is personal and subjective, but it must still maintain a somewhat academic quality and must still be thoroughly and cohesively organized about any aspect of your topic or your experience of working for it from the beginning till the end. The format of reflection writing used for HIPs in the CVS Module in the medical curriculum: (a) Self-critic of their presentation, (b) Description of their experience. The word limit given to students was 500-2000 words.

(4) Facilitators were assigned to do an assessment using the Rubric shared (Appendix 2): Five best presentations (poster or video) are recommended to be posted on the University website and University Twitter/LinkedIn pages. The Best presentation is recommended to be awarded during the student award ceremony day. It was suggested that all the posters could be presented at an Expo as an awareness program for the community. A few examples of the topics chosen for HIPs in the CVS Module are as follows: Role of good sleep and healthy habits prevention of cardiovascular diseases; Prevalence of obesity and hypertension in children taking fast and processed food regularly; Impact of lifestyle modification in the prevention and control of hypertension; Role of screening in prevention of cardiovascular diseases.

HIPs implementation strategies followed in CVS Module are given in Appendix 1 and the rubric used in HIPs for CVS Module is given in Appendix 2.

## Results

**Figure 1:**
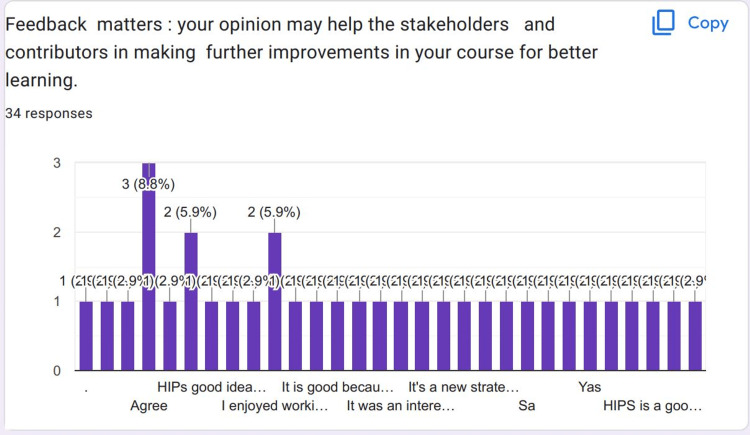
Most of the students strongly agreed that HIPs were a good idea to be included in the CVS Module HIPs: High-impact practices; CVS Module: Cardiovascular Module

**Figure 2:**
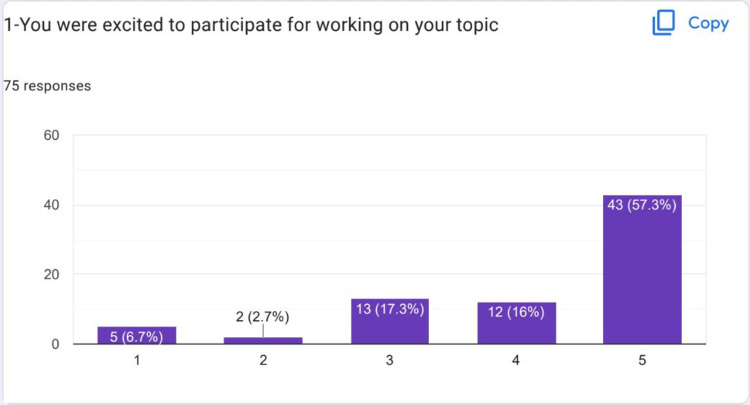
Most of the students strongly agreed that they were excited about their HIPs topic HIPs: High-impact practices

Feedback was received from 75 students. Most of the students who responded were females. Most of the students strongly agreed that HIPs were a good idea to be included in the CVS Module (Figure 1). Most of the students strongly agreed that they were excited about their HIPs topics (Figure 2).

**Figure 3:**
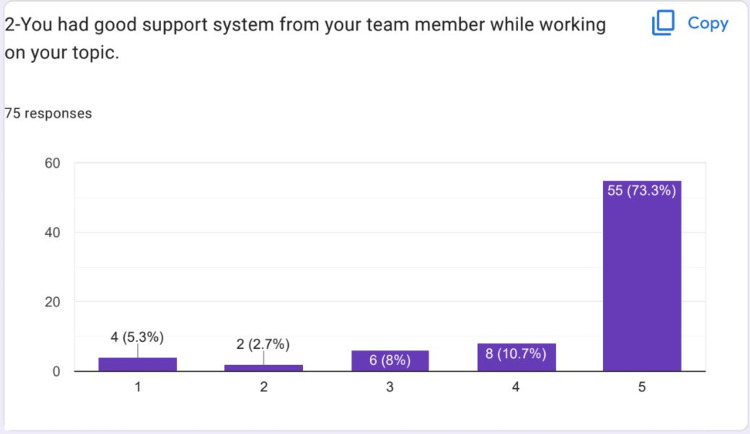
Most of the students strongly agreed that they had a good support system from their team members while working on their HIPs topic HIPs: High-impact practices

**Figure 4:**
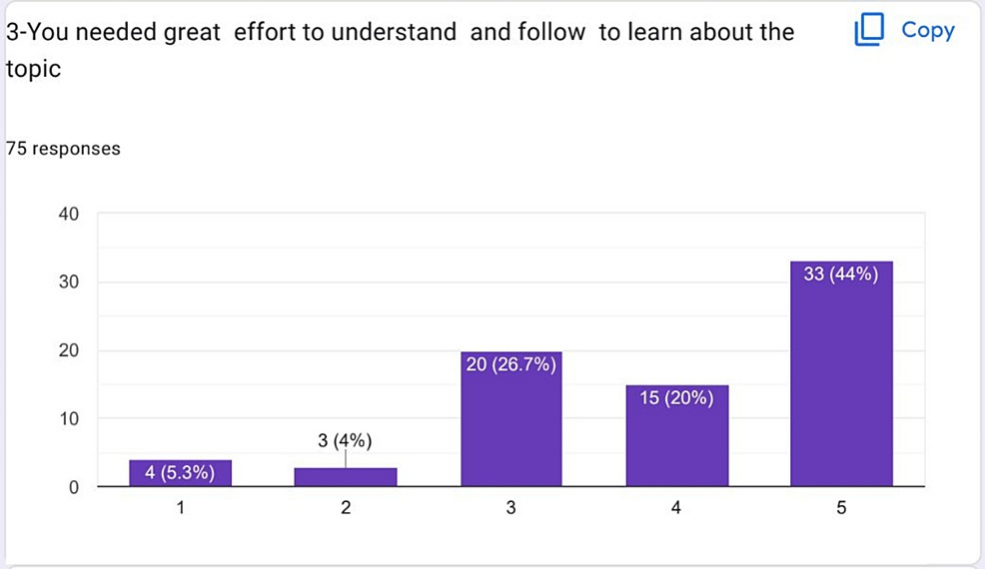
Many students felt that they had to do an effort to understand and learn about their HIPs topic deeply HIPs: High-impact practices

Most of the students strongly agreed that they had a good support system from their team members while working on the HIPs topic (Figure 3). Many students felt that they had to do an effort to understand and be able to learn about their HIPs topic deeply (Figure 4). 

**Figure 5:**
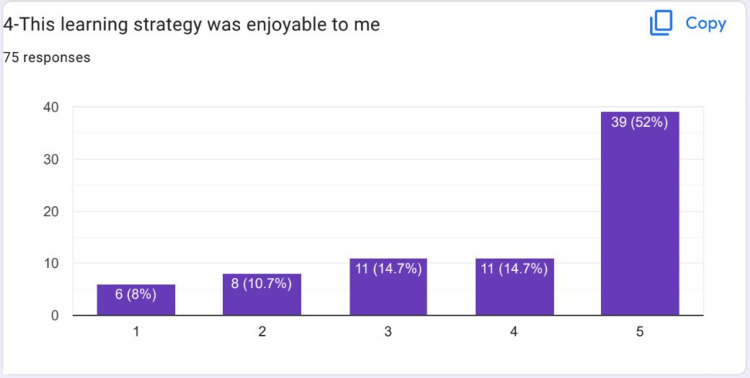
Most of the students strongly agreed that they enjoyed the learning strategy in HIPs HIPs: High-impact practices

**Figure 6:**
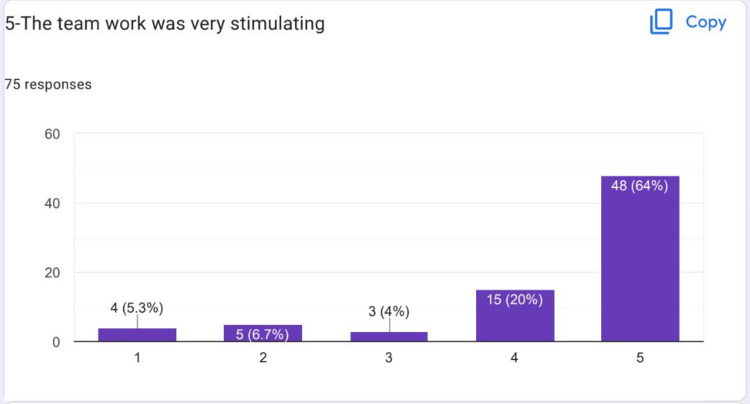
Most of the students strongly agreed that teamwork on HIPs was very stimulating HIPs: High-impact practices

Most of the students strongly agreed that the learning strategy in HIPs was enjoyable to them (Figure 5). Most of the students strongly agreed that teamwork on HIPs was very stimulating (Figure 6). 

**Figure 7:**
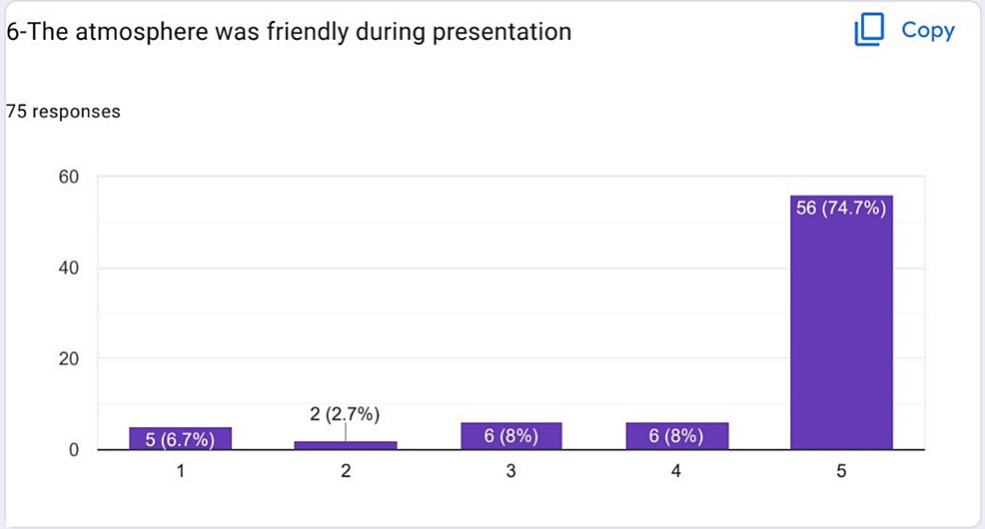
Most of the students strongly agreed that the atmosphere was friendly during HIPs presentations HIPs: High-impact practices

**Figure 8:**
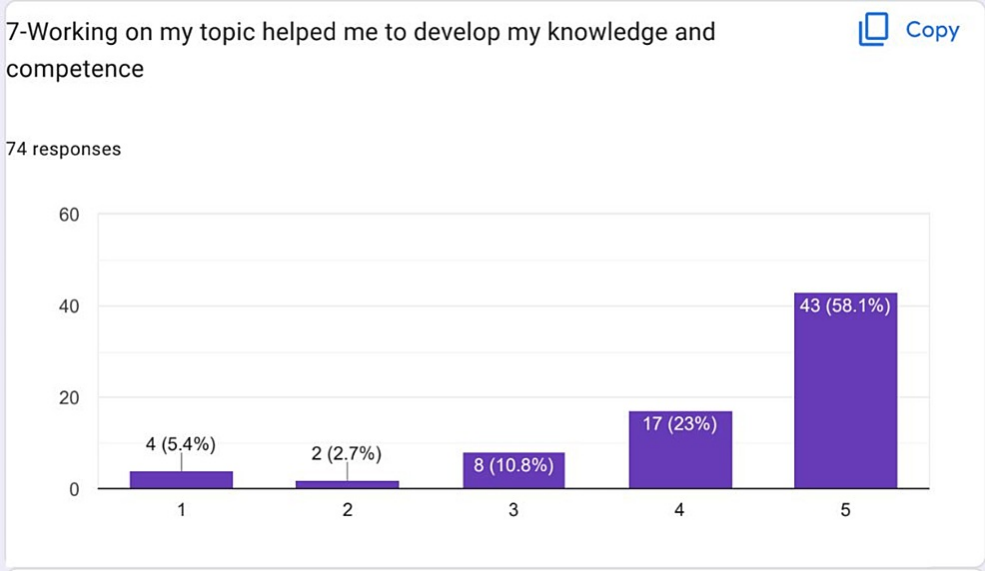
Most of the students strongly agreed that working on their HIPS topic helped them to develop their knowledge and competence

Most of the students strongly agreed that the atmosphere was friendly during the HIPs presentation (Figure 7). Most of the students strongly agreed that working on their HIPs topic helped them to develop their knowledge and competence (Figure 8). 

**Figure 9:**
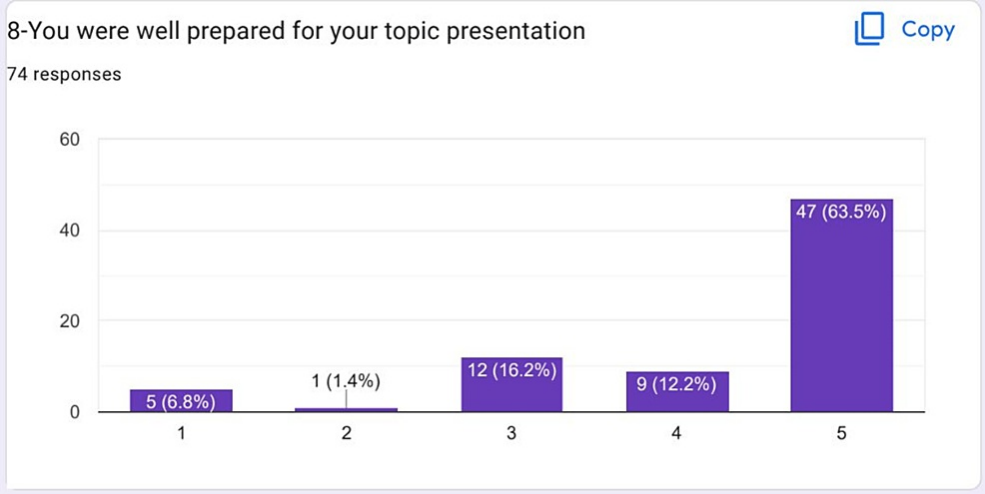
Most of the students strongly agreed that they were well prepared for their topic presentation on HIPs HIPs: High-impact practices

**Figure 10:**
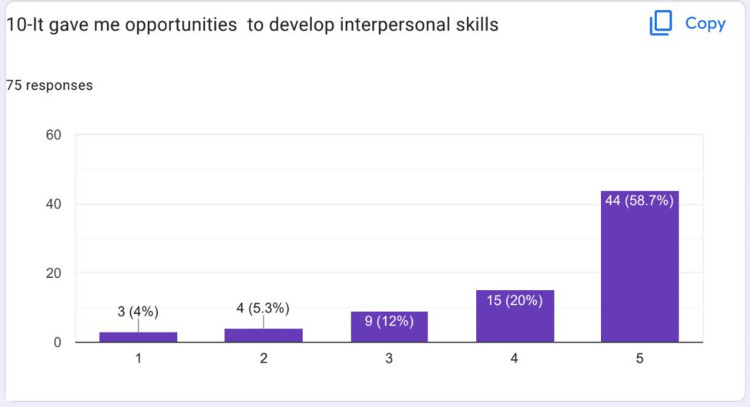
It gave me an opportunity to develop interpersonal skills

Most of the students strongly agreed that they were well prepared for their topic presentation on HIPs (Figure 9). Most of the students strongly agreed that they developed interpersonal skills (Figure 10). 

**Figure 11:**
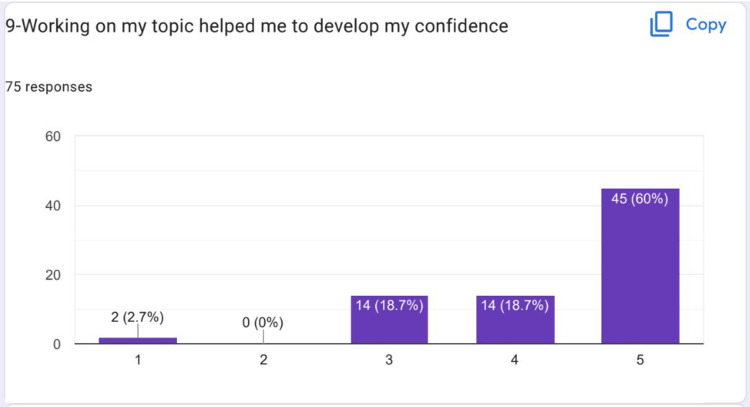
Most students agreed that it helped to develop their confidence

Most Students agreed that it helped to develop their confidence (Figure 11).

From our results, we noticed that students stated that HIPs helped them in an overall improvement in their performance. Also, HIPs experiences may better prepare them for the demands of the real world.

**Figure 12:**
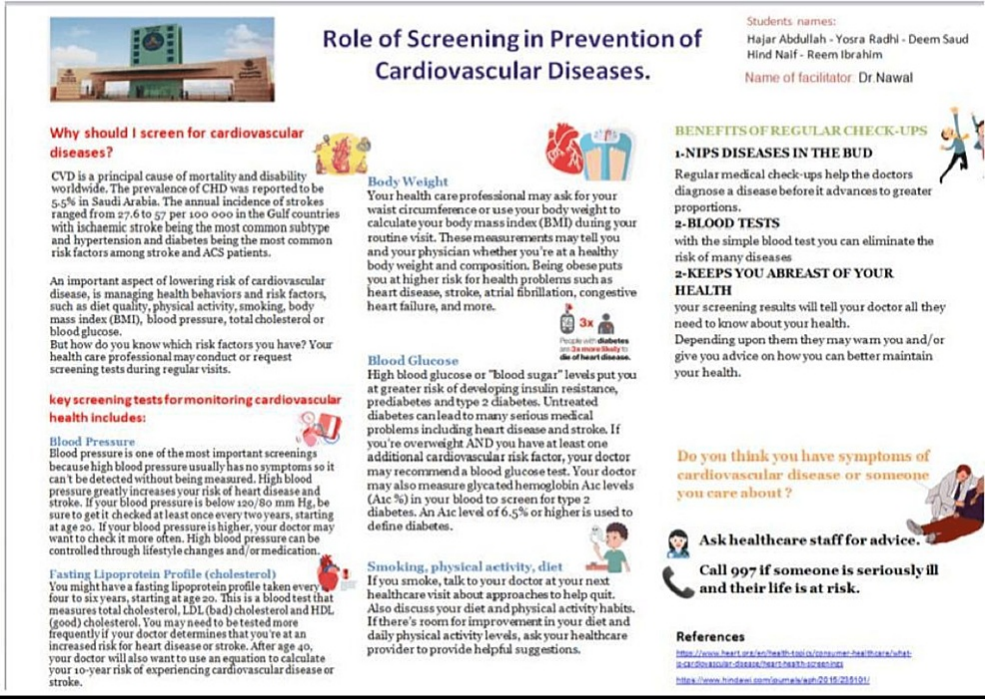
Example of poster made by students for HIP presentation in CVS Module CVS Module: Cardiovascular Module

All groups including all male and female students were evaluated based on the rubric send o them for the poster (Figure 12) and video made on their topics and they scored high marks for meeting the evaluation criteria and fulfilling the idea about implementing HIP for the first time in the medical curriculum in the cardiovascular module.

## Discussion

Teaching strategies in medical education face many difficulties. Saudi Arabian medical education is reforming, but at a gradual pace and with challenges. The majority of their forms focus on curriculum content rather than outlining the fundamental abilities, information, standards, and conduct (outcome-based approach) required of graduate physicians. In their work titled, "Curriculum or syllabus: which are we reforming? ," Implementation of high-impact practices is essential to prepare future health profession students to compete in the world of data-driven science. Yet, few universities have developed high-impact practices and teaching methodologies to impart core competencies to the students in their curriculum [13-14]. Both the number of undergraduate programs providing degrees in health profession education courses and the number of students getting such degrees have grown rapidly. What, if anything, do those who earn these degrees receive training for? What professions, if any, are they preparing for? Is the degree's main goal to get students ready for graduate school or programs for health professionals? Or, is it that "knowing the subject is a crucial component and a necessity for accepting responsibility for developing healthy societies" that justifies the existence of the degree [15]? The effectiveness of efforts to promote community health should be supported by students who have outstanding analytical abilities, in addition to the broad benefits that could result from an increasingly educated population [16-17]. High-impact practices (HIPs) have the potential to improve the requirements, efficacy, and outcomes of student education. Some high-impact strategies are purposefully incorporated into the overarching goals, learning objectives, and curricular competencies for undergraduate degree programs, while others just simply fit in. Nevertheless, careful effort across the phases of curriculum design, course delivery, and program administration is required to maximize the benefits of HIPs for medical students [18-19].

HIPs are still not frequently used in many disciplines, especially in medical educational institutions, despite the mounting evidence that they may be a powerful tool for fostering student learning and engagement. The absence of a conceptual framework to methodically direct study designs and integrate findings, as well as the absence of a medical sciences faculty committed to initiating and implementing an essential pedagogical instruction plan, are two significant barriers to advancement. By adopting HIP to enhance student learning and engagement, our module creates a foundation for multi-disciplinary empirical studies [20].

One earlier study [21] has emphasized their worries that sometimes the changed curricula have not resulted in any evident reforms. To ensure that each effort is focused on internationally recognized standards and goals, the current phase of reform and expansion must also be linked with accreditation and quality assurance methods. Since It is necessary to do systematic planning that takes all potential difficulties into account, hence high impact practices are being introduced to give medical students at par with changing healthcare trends globally.

For example, some teaching and learning methodologies and practices might be better suited to a specific discipline or course; others might be most helpful for students at certain levels; and still, others might simply be more agreeable to the teaching style of a given faculty. Medical schools have a variety of teaching strategies, activities, and teaching tools that can potentially be valued for a given learning objective. However, studies have shown that some teaching strategies seem to have a greater influence than others. Additionally, since both teachers and students are typically stressed for time, it is logical to devote particular attention to teaching strategies that provide "better value for the buck."

Medical colleges in the near future will be encouraged to be more socially accountable if social accountability is made a criterion for the evaluation and assessment of medical institutions [22-24]. Medical schools are divided into three groups based on how much they address community health needs: (a) neutral medical schools, which carry out their duties without much consideration for how they might need to adapt to changing needs of individuals, families, and the community at large; (b) reactive medical schools, which are aware of these needs and respond responsibly; and (c) proactive medical schools. A medical school that takes initiative makes use of its resources to spot possible issues with the healthcare system and works with partners to create and implement solutions [25].

Our initiative to incorporate high-impact practices from the early years of medical students' training may have a great influence on community health. By incorporating HIPs, we aim to improve society’s health status by training competent health professionals who can meet the community's evolving healthcare demands and expectations.

Higher student grade point averages (GPAs), "deep approaches to learning," higher rates of student-faculty interaction, improvements in critical thinking and writing skills, a greater awareness for diversity, and increased student engagement overall are all positively correlated with HIPs. All of the aforementioned points indicate that students participating in HIPs are more likely to finish with high GPAs and demonstrate mastery of critical course skills. In order to be eligible for the designation of "high-impact practices," they must provide the amount of time and effort needed by students. HIPs demand that students devote considerable time and effort to purposeful tasks and require daily decisions that deepen students' involvement in learning as well as their dedication to their course.

Other characteristics of HIPs are as follows- (a) Effective teacher-student interactions: They encourage students to engage in lengthy discussions about key ideas with teachers and their peers. (b) Working with people from various backgrounds: Through interacting with others who are different from them, HIPs "increase the probability that students will deal with diversity, which may challenge students to develop novel ways of thinking to new situations and problems as they work together in parallel with peers on intellectual and practical problems, inside and outside the class, on and off campus." (c) Constant, consistent feedback: HIPs require feedback for improvements and better learning, and “because students perform in close proximity to faculty or peers and work in groups as a team so feedback is almost continuous.” (d) Opportunities to apply concepts in real-world settings: Students have the chance to "see how what they are learning can help the community and may help them in the future to be good doctors" thanks to these possibilities. (e) Reflection and integrative learning opportunities: HIPs "deepen learning, support students in adopting values and beliefs, and help them expand in their abilities for comprehending events and actions by actively participating in them." Students learn more about others and the world at large as a result [26-27], and they can additionally develop the learning tools and comprehend moral principles for the improvement of human health care.

## Conclusions

We have tried to include HIPs in the cardiovascular module for the third-year medical curriculum of MBBS students at Northern Border University. The feedback from students had been encouraging, so we recommend it to be included in all other courses also for the benefit of students to help them remember important concepts about the topics for a longer time and help them to develop critical thinking and deep learning. Also, the Faculty of Medicine at Northern Border University wishes to make advancements in the area of social responsibility, hence it tried to introduce high-impact practices in the medical curriculum. It was a challenge as faculty and students had time constraints still they made an attempt, and it was a success. Except for time management, there was no other challenge stated by students in taking their feedback. However, a transition from the conventional way of teaching methodology to HIPs may be like taking a road to be able to fulfill the full range of social accountability for which the medical college may need to engage with stakeholders more frequently. The following areas need the college's special attention: (a) regional health challenges, not just those involving the health system or services; (b) issues involving the social determinants of health; and (c) cost-effectiveness in both teaching and research.

We used HIPs to solve these issues. First, we aim to supplement written assessments with HIPs in order to improve exam success rates. We also enforce requirements and modify course offers to align with the curriculum roadmap, hence we recommend HIPs to be included in other courses of MBBS as it may foster (a) high achievement rates among students in their courses, (b) help in improving the critical thinking abilities and (c) help to overcome enrolment constraints.

## Appendices

**Table 1:** HIPs implementation strategies followed in CVS Module HIPs: High-impact practices; CVS Module: Cardiovascular module The sequence of steps followed by students in implementing HIPs: (1) Students brainstormed problems related to common topics; (2) Students identified a problem to solve; (3) Students researched the problem; (4) Students performed 1st hand research (interviews, surveys, or focus group); (5) Students designed a solution based on research (brainstorming, objectives, and planning); (6) Students presented problems and solutions; (7) Students reflected on learning and experiences; (8) Students completed HIPs student survey; (9) Students shall organize and facilitate community conversations at exhibitions; (10) Faculty use HIPs rubric for assessing students.

Full Name		ID Number	
Email		Phone Number	
College		Level	
Program		Course Name	
Department		Course Number	
		Number of Students	

**Table 2:** Rubric used in HIPs for CVS Module HIPs: High-impact practices; CVS Module: Cardiovascular module

	Exemplary(2 marks)	Competent (1.5 mark)	Sufficient (1 mark)	Needs improvement (0.5 mark)
Define the problem	Demonstrates the ability to	Demonstrates the ability	Begins to demonstrate	Demonstrates a limited
	construct a clear and insightful	to construct a problem	the ability to construct	ability inidentifying a
	problem statement with evidence	statement with evidence	aproblem statement	problem statement or
	of all relevantcontextual factors.	of mostrelevant factors,	With evidence of most	related contextual
		and problem statement is	relevant factors, but	factors.
		adequately detailed.	problem statement is	
			superficial.	

Propose and evaluate solutions	Proposes one or more solutions contextualized to ethical, logical, and cultural dimensions of the problem. Thorough evaluation considers history of problem, reviews reasoning, examines feasibility and weighs impacts ofsolution.	Proposes one or more solutions indicates comprehension of the problem. Contextualized some factors: ethical, logical, or cultural dimensions of the problem. Adequate evaluation of solutions considers history of problem, reviews reasoning, examines feasibility and weighs impacts of solution.	Proposes one solution that is “off the shelf” rather than individually designed to address the specific contextual factors of the problem. Evaluation of solutions is brief but still considers history of problem, reviews reasoning, examines feasibility of solution, and weighs impacts of solution.	Proposes a solution that is difficult to evaluate because it is vague or only indirectly addresses the problem statement Evaluation of solutions is superficial, but attempts to considers history, reasoning, feasibility, and impacts
Form: Delivery, organization, language/ mechanics	Cohesive organization. Language effective and appropriate to audience. Communication is compelling and polished	Clear, consistent. Language thoughtful and generally supportive. Interesting, speaker comfortable	Organization is intermittently observable. Language understandable but tentative	Organization pattern not observable. Language unclear, minimally supportive, not appropriate to audience Speaker. Distracting and uncomfortable.
Content: Central message Supporting material	Central message is compelling, precisely stated, appropriately repeated, memorable, andstrongly supported with a variety of information or analysis.	Central message is clear and consistent with the supporting material. Appropriate and generally supportive	Central message is basically understandable but is not often repeated andis not memorable. Appropriate but only partially supports	Central message can be deduced, but isnot explicitly stated in the presentation Insufficient supporting materials, minimally supportive.

Implementation Best ideas carried out in Community.	Creativity, innovation, risk- taking, Ideas into actionplanning, management. Implements the solution in a manner that addresses thoroughly and deeply multiple contextual factors of theproblem.	Implements the solutionin a manner that addresses multiple contextual factors of the problem in a surface manner.	Implements the solution in a manner that addresses the problem statement but ignores relevant contextual factors.	Implements the solution in a manner that does not directly address the problem statement.
